# Influence of Recycled Fine Aggregate Content on Properties of Soft Soil Solidified by Industrial Waste Residue

**DOI:** 10.3390/ma15217580

**Published:** 2022-10-28

**Authors:** Anhui Wang, Qiwei Zhan, Wanying Dong, Weiyang Gu, Juanlan Zhou, Zhihong Pan

**Affiliations:** 1 China Construction Industrial & Energy Engineering Group Co., Ltd., Nanjing 210046, China; 2 College of Transportation, Southeast University, Nanjing 211189, China; 3 School of Civil Engineering and Architecture, Jiangsu University of Science and Technology, Zhenjiang 212003, China

**Keywords:** recycled fine aggregate, industrial waste residue, soft soil, solidification

## Abstract

The influence of recycled fine aggregate content on the properties of soft soil solidified by industrial waste residue was systematically studied. First, the addition of recycled fine aggregate may provide skeleton support, which was conducive to improving the solidification properties. Comparing the addition of recycled fine aggregate content and a composite solidification agent separately, the compressive strength increased 48.01 times and 1.32 times, respectively. Second, the composition and quantity of the hydration products were analyzed by X-ray diffraction (XRD) and thermal gravity analysis (TG/DTG). In addition to silicon dioxide and aluminum oxide, a number of new minerals, including hydrated calcium silicate, calcium hydroxide and ettringite, were produced under different recycled fine aggregate contents. The diffraction peak of hydrated calcium hydroxide was weak, which indicated that the crystallinity and relative content was low. The main reason for this was that it was consumed as the activator of the secondary hydration reaction of blast furnace slag. With the increase in recycled fine aggregate content, the total weight loss (hydration products, crystal water, impurities) increased significantly, at rates of 6.9%, 7.0%, 7.2%, 8.8% and 9.7%. The addition of recycled fine aggregate does not change the composition and quantity of the hydration products, and the increased weight loss in this part might be caused by the cement paste attached to the surface of the recycled fine aggregate. Finally, their microstructure was analyzed by scanning electron microscopy (SEM). Larger and more pores appeared in the solidification system with the increase in recycled fine aggregate, and a large amount of ettringite was prepared. An excess in recycled fine aggregate caused more pores, and the negative impact of too many pores exceeded the lifting effect of the aggregate, resulting in the decline of its mechanical properties. Therefore, there was a suitable range for the use of recycled fine aggregate, which was not more than 40%. In conclusion, recycled fine aggregate not only acts as a skeleton to improve solidification strength, but could also realize the comprehensive utilization of waste, which provided a new scheme for solid waste utilization and soft soil solidification.

## 1. Introduction

With the rapid development of the economy and the vigorous promotion of urbanization, the construction industry has rapidly developed as well. However, in the process of urbanization and urban renewal, a large amount of construction waste is generated, including waste sludge and solid waste. At present, most construction waste is directly buried and stacked in the open air; however, the huge amount of construction waste not only occupies a lot of land resources, but also poses a great threat to the ecological environment [1,2,3]. Therefore, how to realize the stable, harmless and resourceful disposal of construction waste has become the top priority in comprehensive environmental management [4,5,6].

Regarding soft soil solidification technology, some fruitful research results have been achieved. Portland cement, lime and industrial waste slag are usually used as solidification materials, and good solidification effects have been obtained. Zhu et al. demonstrated that compressive strength was related to cement content, and that failure strain decreased exponentially according to cement content; with the increase in cement content and age, the stress–strain relationship of solidified soil changed from plasticity to brittleness, and an optimal cement content and age in the overall skeleton structure was identified [7,8]. Ma et al. developed a curing agent composed of magnesium oxide, magnesium chloride and cement to solidify soft soil in order to form a very solid ternary hinge system. It could not only greatly improve the strength of the sludge, but also effectively seal the heavy metal ions in the sludge [9]. Chen et al. confirmed that the compressive strength of solidified sludge was greatly increased after 30% silica fume replaced cement, and the water content and volume shrinkage of the sludge could be effectively reduced. Magnesium potassium phosphate cement and silica fume formed a skeleton structure which could promote the evaporation of water in the sludge and play a key role in sludge reduction [9,10]. However, Portland cement and lime had the disadvantages of high energy consumption and high pollution emissions, which limited their application. There were still some defects in the soft soil solidification of industrial waste residue due to its wide source, multiple types, complex components and unstable nature [11,12,13]. Currently, the research on soft soil solidification mainly focuses on the selection of curing materials, the optimization of the formula of the curing agent, the macro performance and the mechanism of action. This research encountered a bottleneck, and it was difficult to make a substantial breakthrough [3,4,5,14]. Referring to relevant research on concrete, with sand aggregate playing a role of skeleton and filling, the mechanical properties and the durability of concrete were significantly improved [15,16]. Therefore, it was attempted to add sludge to the aggregate in order to improve its solidification performance. However, aggregate resources were extremely scarce, and the price was relatively high. The replacement of natural aggregate by recycled aggregate could solve the cost problem, with the addition of being green, low-carbon and environmentally friendly [17,18,19,20]. Rebeca et al. proved that the mortar with 25% of recycled fine aggregate performed better, which was related to pore structures and their distribution [21]. Amardeep et al. revealed that the municipal solid waste’s incinerated bottom was used as a partial replacement for recycled fine aggregate. This could improve the compressive strength of concrete up to a certain extent, but over-addition led to a detrimental effect on strength [22]. Aline et al. found that the recycling of industrial waste, such as that produced during the processing of ornamental stones, was a relevant procedure to mitigate the possibility of environmental pollution. With the loss of strength in soil–cement blocks following 90 days of immersion cycles, together with a relatively small gain in water absorption, the block composition with 60% of waste was able to meet the norm [23]. The previous research results show that recycled aggregate has been applied in the field of civil engineering, and that it may be feasible in soft soil reinforcement, which should be fully demonstrated [24,25,26,27,28,29,30].

In this paper, the influence of recycled fine aggregate content on the properties of soft soil solidified by industrial waste residue was systematically studied. First, the skeleton filling effect of the recycled fine aggregate was verified, and the influence of recycled fine aggregate content on the compressive strength of solidified soft soil was clarified. Second, the hydration products were analyzed by X-ray diffraction (XRD) and thermal gravity analysis (TG/DTG), and their composition and quantity were determined. Finally, their microstructure was analyzed by scanning electron microscopy (SEM), and the change in hydration products in the presence of the recycled fine aggregate was confirmed.

## 2. Materials and Methods

### 2.1. Materials

**Figure 1:**
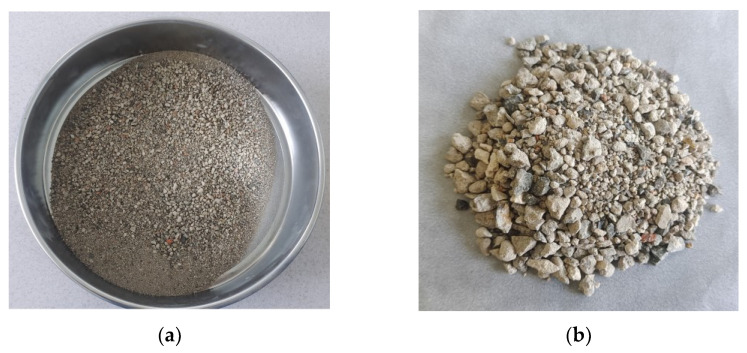
Recycled fine aggregate. (**a**) Original recycled fine aggregate; (**b**) Recycled fine aggregate after screening.

The soft soil was obtained from the Dongliu road reconstruction and expansion project in Qixia District, Nanjing. According to the relevant requirements in the code for highway geotechnical testing (JTG 3430-2020), the particle size, water content, liquid plastic limit and other relevant physical indices of soft soil were measured. The results are shown in Table 1. On the basis of previous tests, each material’s content was composed of 6% of Portland cement, 14% of blast furnace slag and 5% of phosphogypsum. The mass ratio of Portland cement, blast furnace slag and phosphogypsum was 1:2.3:0.8, which was the best formula for a composite solidification agent. The discarded bricks and concrete were crushed by a crusher, and recycled fine aggregate was obtained through screening. The recycled fine aggregate is shown in Figure 1.

### 2.2. Experimental Design

In the previous tests, the properties of soft soil solidified by the best formula of composite solidification agent had been fully demonstrated. In this experiment, it was intended to play the role of skeleton filling of recycled fine aggregate, and further improve the properties of solidified soft soil. In order to improve its properties, recycled fine aggregate was added, and the different contents were 10%, 20%, 30%, 40% and 50% (calculated by dry soil mass). Soft soil, composite solidification agent, recycled aggregate and water were evenly mixed in proportion, and the resulting solution was poured into a cylindrical mold with a diameter of 39.1 mm and a height of 80 mm. In order to make the formed samples as uniform as possible, the mold was placed on the concrete vibration table and vibrated for 40–60 s after pouring each layer of samples, and gas was discharged during vibration. Then, the sample was placed into a standard box for curing. The temperature of the box was 20 ± 2 °C, and the relative humidity was 95 ± 2%. After curing for 24 h, the sample was demolded and placed in the box for further curing. After a specific age, the sample was taken out for macro and micro analysis.

### 2.3. Analytical Method

A uniaxial unconfined compressive strength meter (Keji, Nanjing, China) was used to measure the strength of solidified soft soil. Three samples in each group were tested in parallel, and the average value was recorded as the unconfined compressive strength.

The composition was examined by XRD with Bruker D8-Discover diffractometer(Berlin, Germany) using graphite monochromatized high-intensity Cu Kα radiation (λ = 1.5406 Å). The scanning angle range was from 5° to 90° 2θ with the step at 0.2 s·step^−1^. TG/DTG analysis was obtained from the STA449 F3 thermogravimetric analyzer (Netzsch, Berlin, Germany). The analyses were carried out simultaneously in a nitrogen atmosphere at a heating rate of 10 °C·min^−1^ between room temperature and 1000 °C.

SEM (FEI Company, Amsterdam, The Netherlands) with a GENESIS 60S energy dispersive X-ray spectroscope (EDS) spectroscopy system and a magnification from 5000 to 10,000 was used to examine morphology and to measure the elemental compositions. The accelerating voltage and spot size of the secondary electron detector were 20 kV and 4.0, respectively. The sample was dried to constant weight in the oven, and it was sprayed with a layer of gold before analysis.

## 3. Results and Discussion

### 3.1. Influence of Recycled Fine Aggregate Content on Compressive Strength of Soft Soil

**Figure 2:**
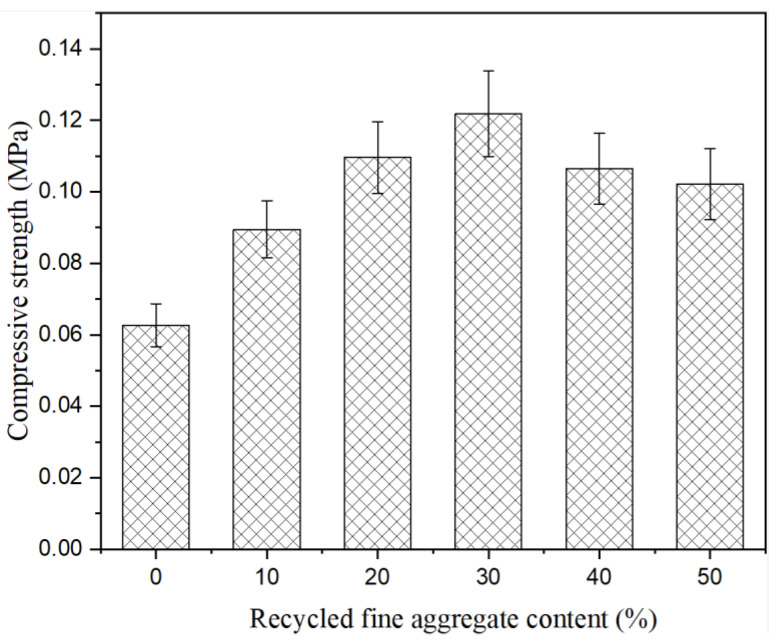
Compressive strength of soft soil solidified without composite solidification agent.

In order to explore the feasibility of recycled fine aggregate as an external material, recycled aggregate was added to soft soil without a composite curing agent, and the influence of recycled fine aggregate content on the compressive strength of soft soil was studied. The results are shown in Figure 2. It can be seen from Figure 2 that recycled fine aggregate content had a significant impact on the compressive strength of soft soil solidified without a composite solidification agent. With the increase in recycled fine aggregate content, the compressive strength of soft soil increased first and then decreased. When the recycled aggregate content was of 0%, 10%, 20%, 30%, 40% and 50%, the compressive strength of the soft soil was 0.06 MPa, 0.09 MPa, 0.11 MPa, 0.12 MPa, 0.11 MPa and 0.10 MPa, respectively. Compared with an absence of recycled fine aggregate, the compressive strength increased by 42.45%, 74.53%, 95.43%, 69.56% and 63.29%, respectively, when recycled fine aggregate content was added at 10%, 20%, 30%, 40% and 50%. When the recycled fine aggregate content was of 30%, the solidification effect on the soft soil was superior. The soft soil was characterized by “three high and two low”, namely, high water content, high porosity, high compressibility, low strength and low permeability. The addition of recycled fine aggregate could provide skeleton support, which was conducive to improving the solidification properties. The above research results show that the technical scheme of adding recycled fine aggregate to improve the solidification effect on soft soil was feasible.

**Figure 3:**
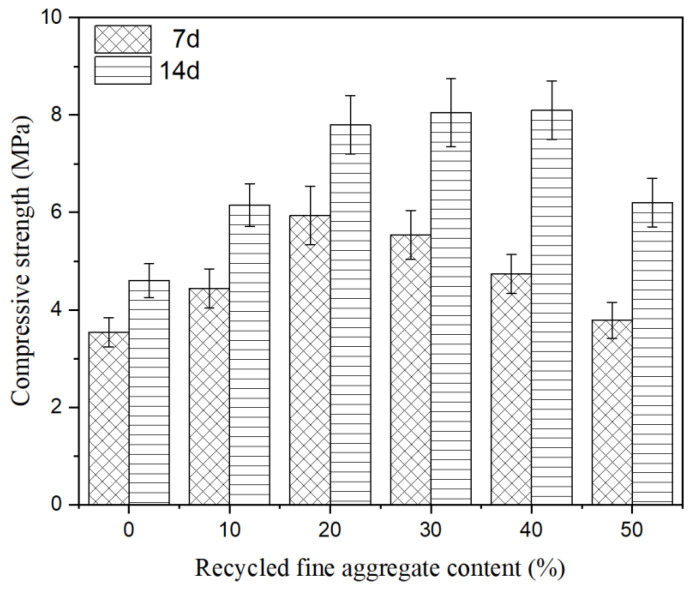
Compressive strength of soft soil solidified by composite solidification agent.

It was feasible to use recycled fine aggregate as an additional material to solidify soft soil, but the solidification effect was not significantly improved by adding recycled aggregate alone, as the compressive strength remained at a low level. The technical scheme of the composite solidification agent cooperating with recycled fine aggregate to improve the solidification properties was designed in the test, and the influence of recycled fine aggregate content on compressive strength was studied. The results are shown in Figure 3. Recycled fine aggregate content had a great impact on the solidification properties of recycled fine aggregate combined with a composite solidification agent. When the solidification age was of 7 days and the recycled fine aggregate content was of 20%, the solidification effect was the best, and the compressive strength increased up to 5.93 MPa. Comparing the addition of recycled fine aggregate and a composite solidification agent separately, the compressive strength increased 48.01 times and 1.32 times, respectively. With the extension of the solidification age, the solidification effect was further improved. When the solidification age was of 14 days, the compressive strength was 8.43 MPa. In conclusion, recycled fine aggregate not only acts as a skeleton to improve the solidification strength, but can also realize the comprehensive utilization of waste.

### 3.2. Composition of Soft Soil Solidified by Recycled Fine Aggregate and Composite Solidification Agent

**Figure 4:**
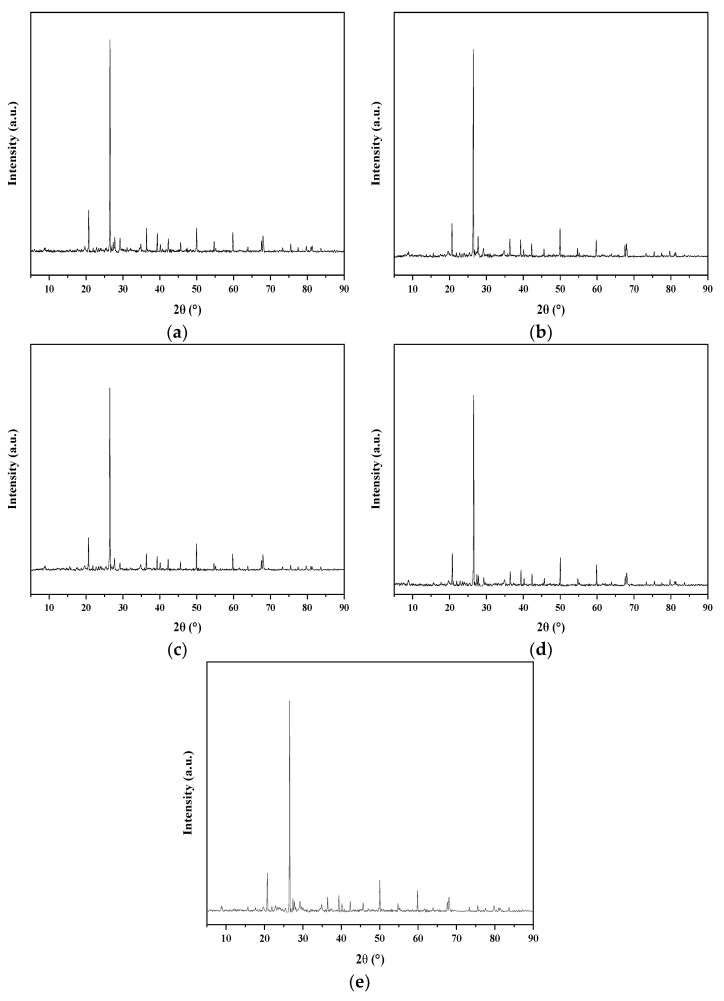
XRD spectra of sample under different recycled fine aggregate contents: (**a**) 10% recycled fine aggregate; (**b**) 20% recycled fine aggregate; (**c**) 30% recycled fine aggregate; (**d**) 40% recycled fine aggregate; (**e**) 50% recycled fine aggregate.

Recycled fine aggregate played a skeleton role in solidified soft soil and, theoretically, it had no significant influence on the composition of the mineral products. This content was systematically and deeply verified by XRD, and the results are shown in Figure 4. MDI JADE 5.0 was used to conduct phase retrieval of XRD spectrogram. Characteristic diffraction peaks of silicon dioxide and aluminum oxide were found under different recycled aggregate contents. The diffraction peaks were strong and clean, which represent the main components of soft soil. In addition, a number of new minerals, including hydrated calcium silicate, calcium hydroxide and ettringite, were produced under different recycled fine aggregate contents. The diffraction peak of hydrated calcium hydroxide was weak, which indicated that the crystallinity and relative content was low. The main reason for this was that it was consumed as the activator of the secondary hydration reaction of blast furnace slag [31,32,33,34,35]. Compared to the composite solidification agent without recycled fine aggregate, the addition of recycled fine aggregate did not change the hydration reaction of various substances nor the type of hydration products.

### 3.3. Hydration Products Quantity of Soft Soil Solidified by Recycled Fine Aggregate and Composite Solidification Agent

**Figure 5:**
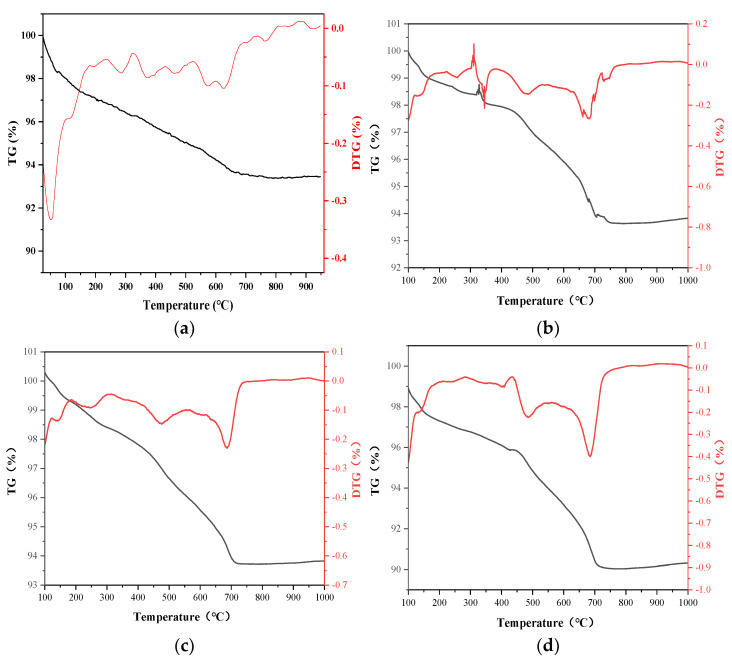

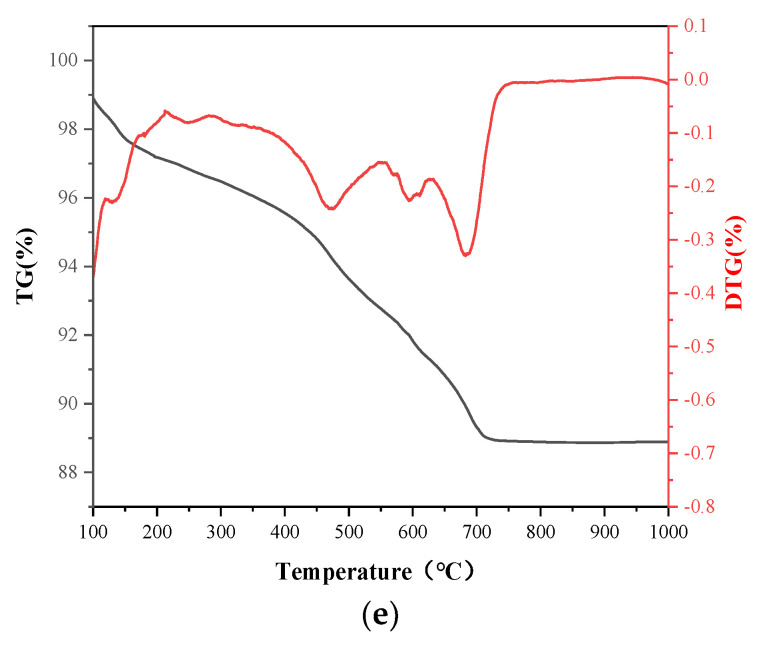
Thermal decomposition of sample under different recycled fine aggregate contents: (**a**) 10% recycled fine aggregate; (**b**) 20% recycled fine aggregate; (**c**) 30% recycled fine aggregate; (**d**) 40% recycled fine aggregate; (**e**) 50% recycled fine aggregate.

The addition of recycled fine aggregate did not change the composition of the hydration products, and the hydration products’ quantity was further studied by TG/DTG (Figure 5). Figure 5 shows that the weight loss of soft soil varied greatly with different recycled fine aggregate contents. With the increase in recycled fine aggregate content, the total weight loss (hydration products, crystal water, impurities) increased significantly, at rates of 6.9%, 7.0%, 7.2%, 8.8% and 9.7%. When recycled fine aggregate content was of less than 30%, the weight loss was essentially equivalent to that when the composite solidification agent (6% cement + 12% blast furnace slag + 3% phosphogypsum) was added separately, indicating that the hydration products’ quantity had not been changed by the addition of recycled fine aggregate. When the recycled fine aggregate content was of 40% and 50%, the overall weight loss increased. Previous studies had confirmed that the addition of recycled fine aggregate would not change the composition and quantity of the hydration products, and the increased weight loss in this part may be caused by the cement paste attached to the surface of the recycled fine aggregate. The decomposition of calcium silicate hydrate, calcium hydroxide and other substances in the cement paste resulted in an increased weight loss. At a low content, the influence on weight loss was not evident due to the low overall content and relatively few cement stones mixed in the recycled fine aggregate. However, with the increase in content, the influence of cement stones mixed in the recycled fine aggregate on weight loss behavior was more evident.

### 3.4. Microstructure of Soft Soil Solidified by Recycled Fine Aggregate and Composite Solidification Agent

**Figure 6:**
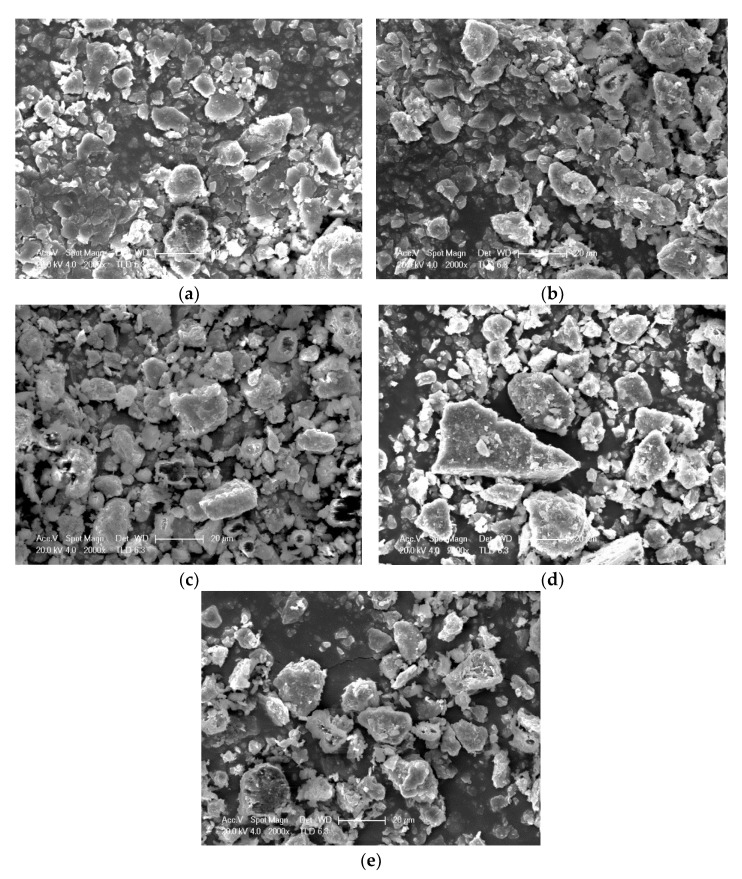
Microstructure of samples under different recycled fine aggregate contents: (**a**) 10% recycled fine aggregate; (**b**) 20% recycled fine aggregate; (**c**) 30% recycled fine aggregate; (**d**) 40% recycled fine aggregate; (**e**) 50% recycled fine aggregate.

The microstructure of samples under different recycled fine aggregate contents are shown in Figure 6. It can be seen from Figure 6 that recycled fine aggregate content had a significant impact on microstructure. When the recycled fine aggregate content was of 10%, soft soil in the solidification system accounted for the majority of it, because the recycled fine aggregate content was small and the soft soil particles were cemented to a very dense structure under the cementation of the hydration products (Figure 6a). As shown in Figure 6b–d, it was difficult for a constant amount of hydration products to wrap, fill and cement more recycled fine aggregate particles with the increase in recycled aggregate content, leading to the structural compactness of soft soil decreasing gradually, while the pore characteristics increased significantly. When the recycled fine aggregate content was of 50%, more and larger pores appeared in the solidification system with the increase in recycled fine aggregate (Figure 6e). These pores provided sufficient space for the formation of the hydration products, and the crystals could grow freely without restriction, forming a large number of needle-like and rod-like products, which were ettringite [36,37,38,39,40]. This also confirmed that calcium hydroxide formed by cement hydration and industrial waste phosphogypsum fully stimulated the secondary hydration reaction of blast furnace slag, and a large amount of ettringite was prepared. The mechanical properties were closely related to its microstructure. Generally speaking, the denser the structure was, the better the mechanical properties were. When the recycled fine aggregate content was of 10%, the solidified soft soil structure was dense, and the compressive strength was 6.19 MPa after 14 days. When the recycled fine aggregate content was of 40%, the structural compactness of the solidified soft soil decreased. However, the compressive strength after 14 days was 8.43 MPa. The research results again show that recycled fine aggregate played a skeleton role and improved the mechanical properties. When the recycled fine aggregate content was of 50%, compressive strength decreased to 5.81 MPa after 14 days. An excess of recycled fine aggregate caused more pores, and the negative impact of too many pores exceeded the lifting effect of the aggregate, resulting in the decline of its mechanical properties. Therefore, there was a suitable range for the use of recycled fine aggregate, and its amount should not be too large.

## 4. Conclusions

In this paper, the influence of recycled fine aggregate content on the properties of soft soil solidified by industrial waste residue was systematically studied. First, the addition of recycled fine aggregate provided skeleton support, which was conducive to improving the solidification properties. Comparing it to the addition of recycled fine aggregate and a composite solidification agent separately, the compressive strength increased 48.01 times and 1.32 times, respectively. Second, the composition and quantity of the hydration products were analyzed by XRD and TG/DTG. In addition to silicon dioxide and aluminum oxide, a number of new minerals, including hydrated calcium silicate, calcium hydroxide and ettringite, were produced under different recycled fine aggregate contents. The diffraction peak of hydrated calcium hydroxide was weak, which indicated that the crystallinity and relative content was low. The main reason for this was that it was consumed as the activator of the secondary hydration reaction of blast furnace slag. With the increase in recycled fine aggregate content, the total weight loss (hydration products, crystal water, impurities) increased significantly, at rates of 6.9%, 7.0%, 7.2%, 8.8% and 9.7%. The addition of recycled fine aggregate did not change the composition and quantity of the hydration products, and the increased weight loss in this part may be caused by the cement paste attached to the surface of the recycled fine aggregate. Finally, its microstructure was analyzed by SEM. More and larger pores appeared in the solidification system with the increase in recycled fine aggregate, and a large amount of ettringite was prepared. An excess of recycled fine aggregate caused more pores, and the negative impact of too many pores exceeded the lifting effect of the aggregate, resulting in the decline of its mechanical properties. Therefore, there was a suitable range for the use of recycled fine aggregate, which was not more than 40%. In conclusion, recycled fine aggregate not only acts as a skeleton to improve the solidification strength, but could also realize the comprehensive utilization of waste, which provided a new scheme for solid waste utilization and soft soil solidification.

## Figures and Tables

**Table 1:** Physical properties of recycled fine aggregate.

Water Content (%)	Liquid Limit (%)	Plastic Limit (%)	Plasticity Index	Maximum Dry Density (g/cm^3^)	Clay Content (%)	Organic Matter Content (%)	pH
45.6	37.2	22.9	14.3	1.82	46	1.72	6.5

## Data Availability

Not applicable.
